# Supplementary material for: Association between Plasminogen Activator Inhibitor-1 and Osimertinib Tolerance in EGFR-Mutated Lung Cancer via Epithelial–Mesenchymal Transition
Source: Cancers (Basel). 2023 Feb 8;15(4):1092. doi: 10.3390/cancers15041092 (PMC9954529; doi:10.3390/cancers15041092)
Supplement: Supplementary file 1 [file cancers-15-01092-s001.zip › Supplementary_figure.pdf]

Supplementary figures

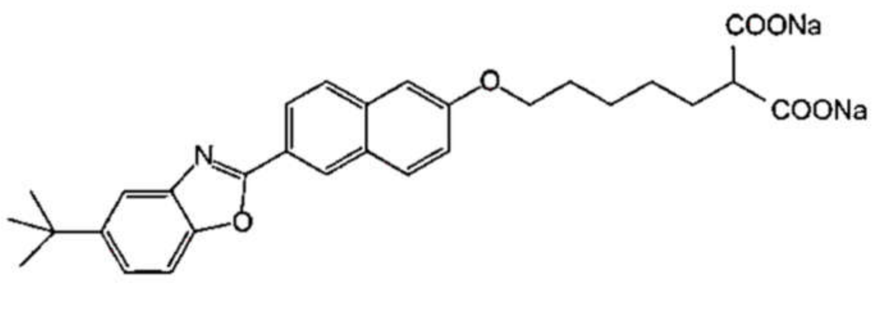

Figure S1
Structure of SK-216

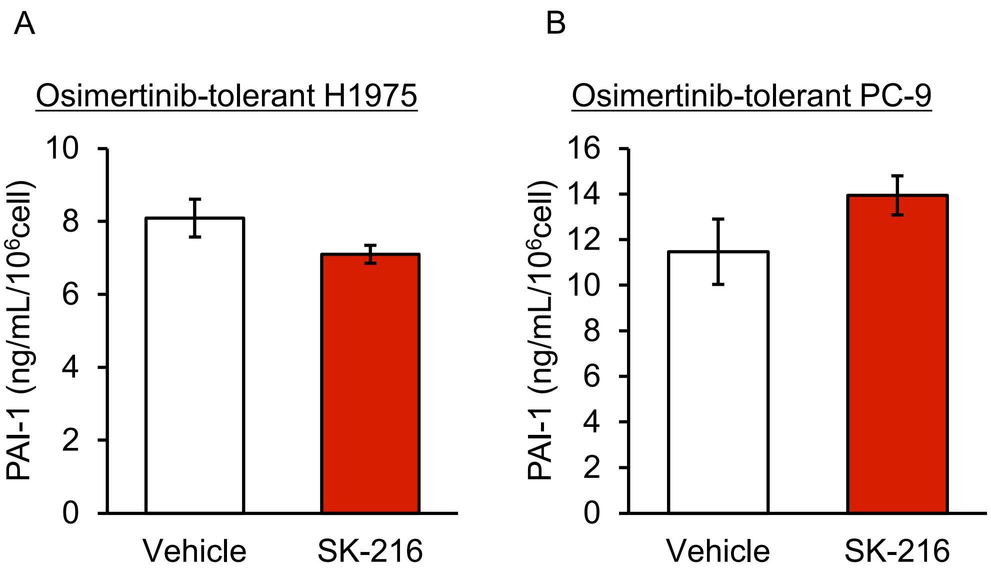

Figure S2

Concentration of PAI-1 protein in the culture medium of (A) osimertinib-tolerant PC-9 and (B) H1975 cells treated with (red) or without (white) SK-216. Mean concentrations are shown with standard error bars (n=5). There were no significant differences between the SK-216 treated and untreated groups (Student's *t*-test).

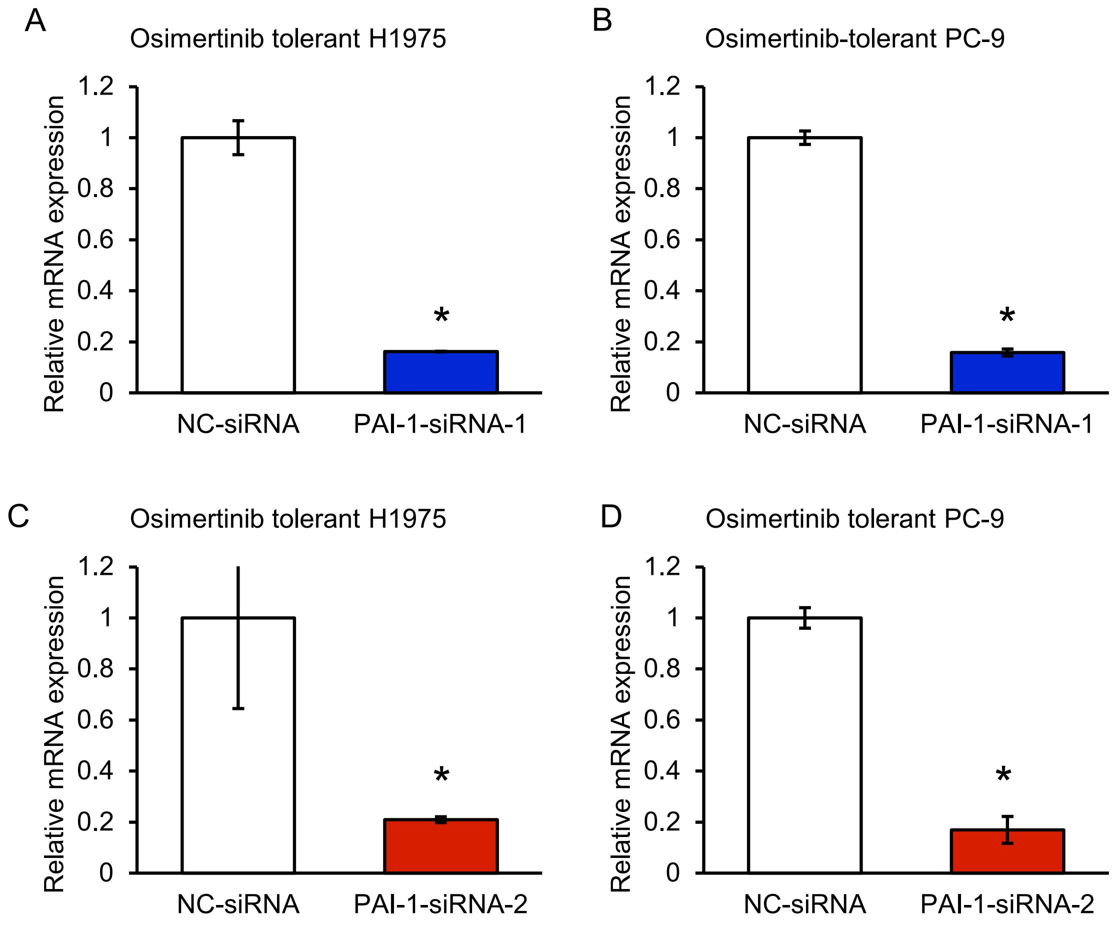

Figure S3

Evaluation of the knockdown efficiency of PAI-1 in (A, C) Osimertinib tolerant H1975 cells and (B, D) PC-9 cells. Cancer cells were exposed to PAI-1-siRNA (PAI-1-siRNA-1 and PAI-1-siRNA-2) to knockdown PAI-1 expression, as indicated in Materials & Methods and compared to the negative control siRNA (NC-siRNA). The data for PAI-1-siRNA-1 and PAI-1-siRNA-2 are shown for the two siRNA reagents described in the Materials and Methods section, s10013 and s10014, respectively. PAI-1 mRNA expression is shown 48 hours after siRNA administration in each cell. Relative expression level of PAI-1 is shown with standard error bars (n=3). *$p<0.05$ compared with NC-siRNA group (Student's t-test).

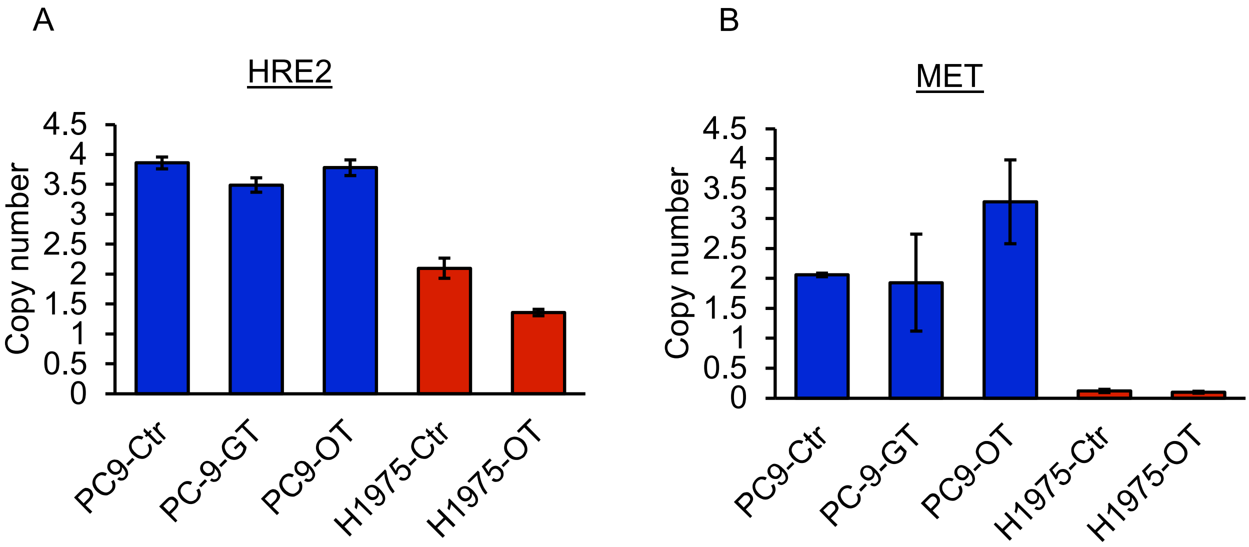

Figure S4

Analysis of the gene copy number of MET and HER2

PCR analysis of the gene copy number of (A) MET and (B) HER2 in EGFR-TKIs tolerant PC-9 and H1975 cells compared to no-treatment controls. Mean copy numbers are shown with SE error bars (n=3), Ctr; Control cells, GT; gefitinib-tolerant cells, OT; Osimertinib-tolerant cells.

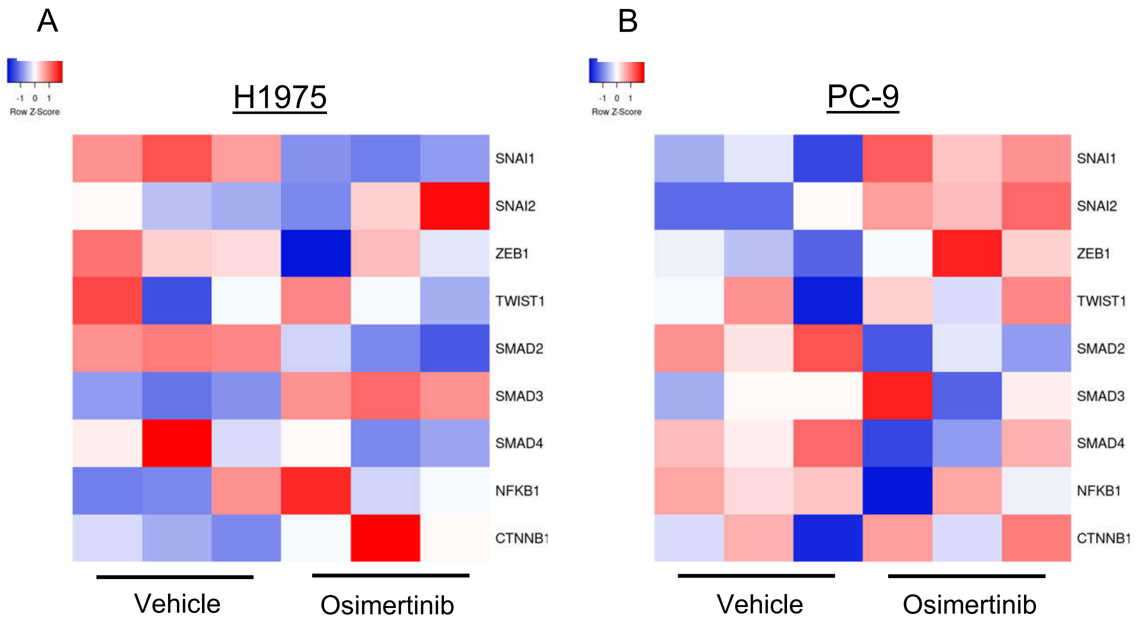

Figure S5

The genes expression involved in TGF-β induced EMT pathway b in the microarray data

The expression of genes constituting the TGF-β induced EMT signaling pathway between vehicle control and osimertinib-tolerant cell groups in (A) PC-9 and (B) H1975 in the heatmap. Each column assigns each sample, and each row assigns each gene.

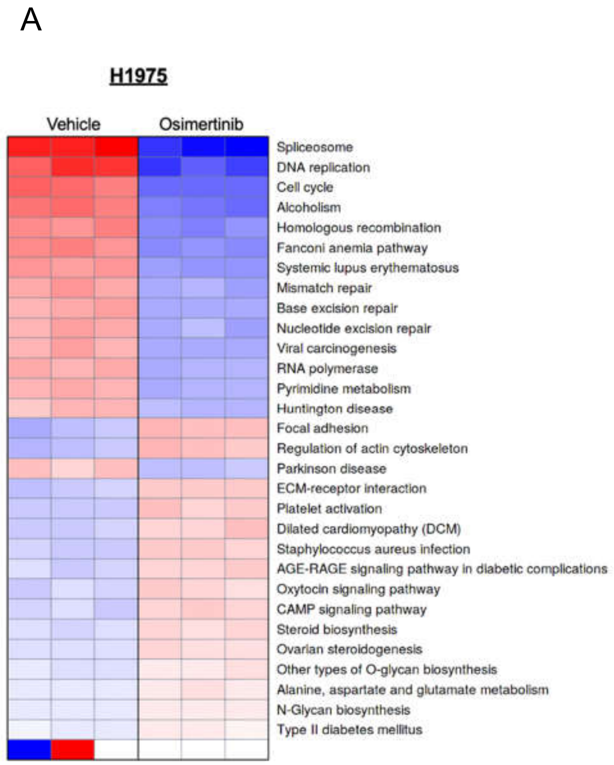

Figure S6

A comprehensive evaluation of mRNA expression via microarrays was performed for osimertinib-tolerant and vehicle control (A) H1975 and (B) PC-9 cells, and pathway analysis was performed. The heatmap shows the results of the pathway analysis from the parametric gene set enrichment analysis of the KEGG gene sets. The top 30 pathways with expression changes in osimertinib-tolerant cells compared with the untreated control are shown. Activated pathways are shown in red, and suppressed pathways are shown in blue.

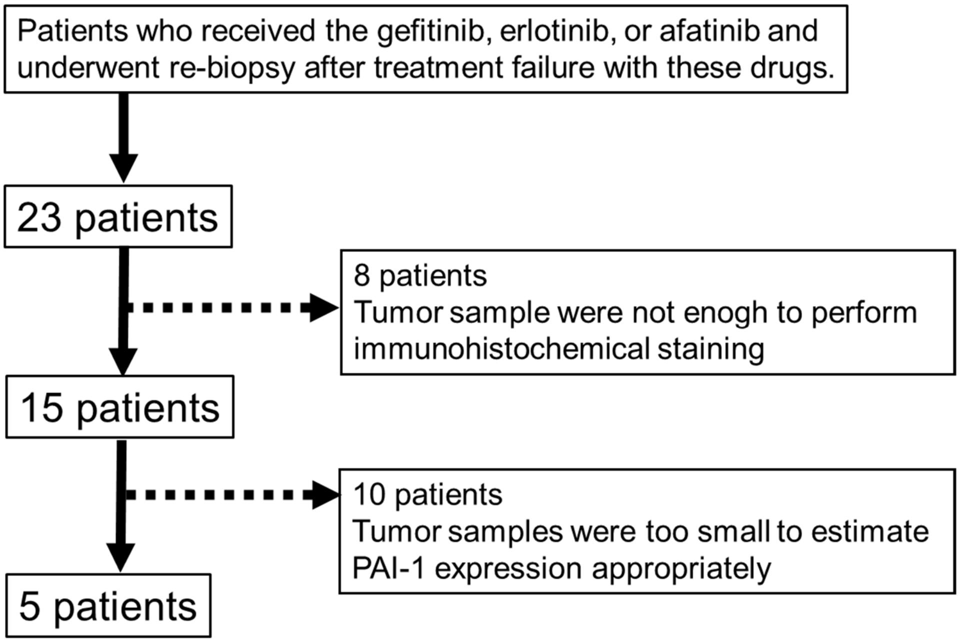

Figure S7

Flowchart of participants

(A) Western blotting image for Figure1 E

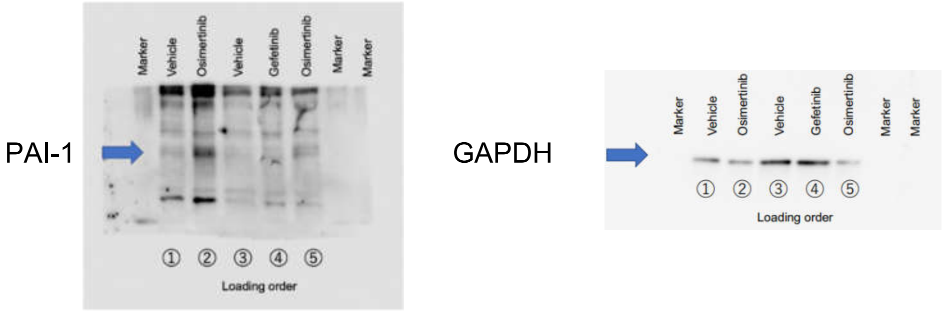

(B) Western blotting image for Figure1 F

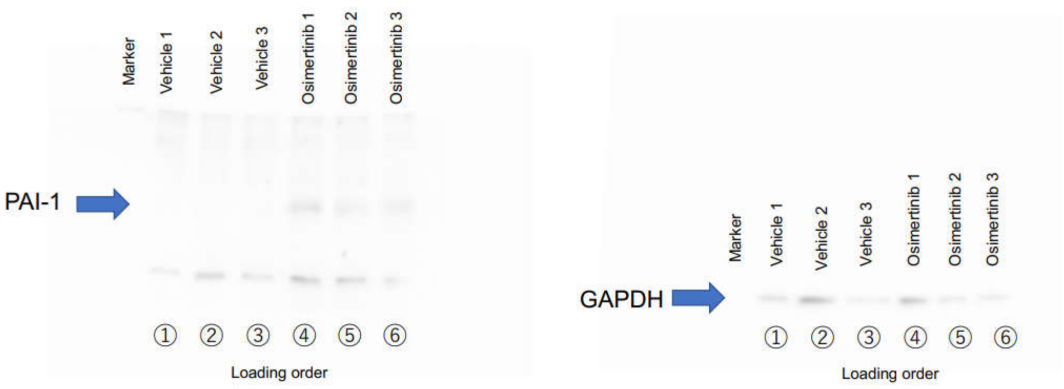

(C) Western blotting image for Figure1 G

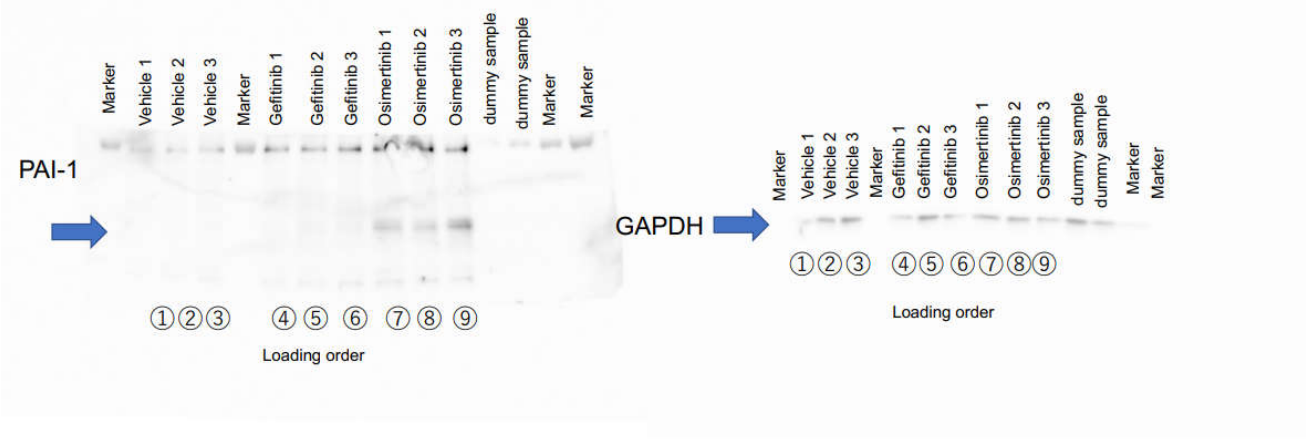

Figure S8
Western blot figures
