# Supplementary material for: Association between Plasminogen Activator Inhibitor-1 and Osimertinib Tolerance in EGFR-Mutated Lung Cancer via Epithelial–Mesenchymal Transition
Source: Cancers (Basel). 2023 Feb 8;15(4):1092. doi: 10.3390/cancers15041092 (PMC9954529; doi:10.3390/cancers15041092)
Supplement: Supplementary file 1 [file cancers-15-01092-s001.zip › Supplementary_methods.pdf]

## Supplementary Methods

### Isolation and culture of cancer cells obtained from a patient

Approximately 300 mL of the patient's pleural fluid was collected and centrifuged (1300 rpm, 5 min, 4°C; all subsequent centrifugations were performed under these conditions). The supernatant was removed, and the pellet was resuspended with PBS, followed by centrifugation again. Only buffy coats were collected in a centrifuge tube, resuspended with PBS, and centrifuged. After the removal of the supernatant, the sample was resuspended in an appropriate amount of Red Blood Cell lysis Buffer (Merck, Darmstadt, Germany) and incubated at room temperature for 6 min. After the removal of the supernatant, the sample was washed with PBS and centrifuged. This process was repeated twice. The remaining components were suspended with culture medium (RPMI-1640 with 10% fetal bovine serum) and seeded into 6-well plates. After 4–5 passages, almost only cancer cells remained. The cells were cultured in RPMI-1640 medium supplemented with 10% fetal bovine serum, penicillin, and streptomycin (100 U/mL and 100 µg/mL, respectively) under a humidified 5% $CO_2$ atmosphere at 37°C in an incubator.

### Quantitative real time PCR (qRT-PCR)

The primers used were as follows: PAI-1 (Hs01126606_m); E-cadherin (Hs01023894_m1); N-cadherin (Hs00983056_m1); fibronectin1 (Hs01549976_m1); α-SMA (Hs00426835_g1); SOS (Hs00362308_m1); HRAS (Hs00978051_g1); KRAS (Hs00364284_g1); Ral (Hs00800233_s1); CDC42 (Hs00741586_mH); SRC (Hs01082246_m1); RhoGAP (Hs00534180_m1); RhoA (Hs00357608_m1); ROCK (Hs00178463_m1), MLC (Hs00853081_g1), and β-actin (4352935E, 4352341E).

All of these reagents are from Thermo Fisher Scientific.

### *EGFR* T790M, C797S mutation, gene copy number analyses

The copy number ratio of the target gene to RNase P (Hs01126606_m1) was then calculated. The primers used were MET (Hs01432482_cn) and HER2 (Hs00817646_cn)

All of these reagents are from Thermo Fisher Scientific.
